# Identification of RNA helicases in human immunodeficiency virus 1 (HIV-1) replication – a targeted small interfering RNA library screen using pseudotyped and WT HIV-1

**DOI:** 10.1099/vir.0.000092

**Published:** 2015-06

**Authors:** Claire A. Williams, Truus E. M. Abbink, Kuan-Teh Jeang, Andrew M. L. Lever

**Affiliations:** Department of Medicine, University of Cambridge, Cambridge, UK

## Abstract

Central to the development of new treatments for human immunodeficiency virus 1 (HIV-1) is a more thorough understanding of the viral life cycle and the cellular cofactors upon which this depends. Targeting cellular proteins and their interaction with HIV-1 has the potential to reduce the problem of emerging viral resistance to drugs as mutational escape is more difficult. We performed a short interfering RNA (siRNA) library screen targeting 59 cellular RNA helicases, assessing the effect on both viral capsid protein production and infectious virion formation. Five RNA helicases were identified which, when knocked down, reproducibly decreased infectious particle production: DDX5, DDX10, DDX17, DDX28 and DDX52. Two of these proteins (DDX5 and DDX17) have known roles in HIV-1 replication. A further helicase (DDX10) was a positive hit from a previous genome-wide siRNA screen; however, DDX28 and DDX52 have not previously been implicated as essential cofactors for HIV-1.

Given the high propensity of human immunodeficiency virus 1 (HIV-1) to mutate, drug resistance is a serious and growing threat, necessitating the ongoing development of new anti-retroviral agents (Pennings, 2013). One possible tactic to address this problem is to design antiviral agents targeting non-mutable cellular proteins required for viral replication. To date, three genome-wide short interfering RNA (siRNA) library screens have been performed to identify cellular factors required for HIV-1 replication (Brass *et al.*, 2008; König *et al.*, 2008; Zhou *et al.*, 2008). Between them, these three studies identified >800 genes as potentially involved in HIV-1 replication. However, a meta-analysis revealed a low (although statistically significant) overlap between the studies, with just three genes positive in all screens (Bushman *et al.*, 2009). Possible reasons for this low overlap include different siRNA design, different experimental timings, different viral strains, focus on different stages of the viral life cycle and different measures of cell toxicity.

Amongst the cellular proteins identified by the three genome-wide siRNA screens were eight RNA helicases. The RNA helicases have a wide range of functions and are crucial for almost every aspect of RNA metabolism (Bleichert & Baserga, 2007). In addition to those identified by siRNA screens, a number of helicases have been shown to be involved in various stages of HIV replication (Chen *et al.*, 2013). RNA helicases have attracted attention as potential antiviral drug targets; several groups have developed compounds to inhibit DDX3, a helicase required for Rev-mediated nuclear export, providing an important proof of principle that RNA helicases could be a valuable therapeutic target (Maga *et al.*, 2008, 2011; Yedavalli *et al.*, 2008).

We used a library consisting of three independent siRNAs against 59 RNA helicases to identify additional helicases with a role in HIV-1 replication and identify potential novel antiviral targets. Our focused RNA helicase siRNA library allowed for multiple rounds of screening in different formats using individual siRNAs rather than siRNA pools, hopefully allowing us to reconcile differences seen in the results of the genome-wide siRNA screens.

The first round of screening was performed in triplicate experiments each in triplicate wells in 96-well plates in HeLa cells to overcome difficulties associated with achieving effective siRNA-mediated knockdown in T-cells. We initially used a pseudotyped HIV-1 system, co-transfecting cells with an envelope-deleted HIV-1 plasmid, a vesicular stomatitis virus (VSV) glycoprotein plasmid and a specific siRNA using Lipofectamine 2000 (Invitrogen). At 96 h post-transfection, samples were collected for capsid p24 (CA-p24) ELISA, intracellular CA-p24 ELISA and assessment of infectious virion production. The infectivity assay utilized TZM-bl cells – a HeLa-derived cell line expressing the receptors necessary for HIV infection and a luciferase gene under the control of a HIV-1 LTR. The assay was optimized to ensure the luminescence produced in response to infection was within the linear range. Included in each experiment were a non-targeting negative control siRNA (siCon) and siRNA against DDX3 as a positive control (siDDX3).

siRNAs were considered to be of interest if they decreased all three measured parameters by >20 %. Also of interest were siRNAs which had no effect on intracellular CA-p24, but did decrease extracellular CA-p24 and infectious virion production, as this could represent a viral assembly defect. Of the 177 siRNAs screened in the first round, 43 fulfilled these criteria. The remaining siRNAs either resulted in no change in the measured parameters or the results were too variable to be reliably interpreted (coefficient of variance >30 % for two of the three measured parameters or >50 % for one parameter). We considered a particular helicase to be of interest if two of the three siRNAs against it resulted in a positive phenotype or if one siRNA gave a positive phenotype and the others were too variable to be interpreted. Overall, 16 of the 59 helicases were taken forward to the second round of screening.

In the second round of screening, the siRNAs of interest were screened similarly in triplicate utilizing the pseudotyped virus system in HeLa cells in 24-well plates with the same three measures of viral replication assessed. Overall, the results in this plate format were less variable than seen in 96-well plates, with good reproducibility between the two plate formats. Only six of the 48 siRNAs rescreened gave a different result in the second round of screening compared with the first round. All helicases taken forward to the final stage of screening had reproducible results with at least two of the three siRNAs. We selected the eight helicases whose knockdown had the largest impact on viral replication for further study.

To determine whether the results seen were specific to the pseudotyped virus system, the siRNAs against the helicases of interest (DDX5, DDX10, DDX17, DDX18, DDX19, DDX28, DDX52 and DDX56) were screened again in triplicate experiments in both 96- and 24-well plates using WT HIV-1 (pLAI). Overall, the effects seen with the WT virus in 96-well plates were less marked than seen with the pseudotyped virus. This may be due to WT viral replication being more robust than pseudotyped replication or effects on the VSV component. The difference between the two systems was most notable in the case of DDX18, DDX19 and DDX56, which were therefore not studied further. The remaining 15 siRNAs were screened again in 24-well plates using WT HIV and a sequential transfection approach, whereby the siRNAs were transfected initially, followed 24 h later by the viral plasmid. The results of the supernatant CA-p24 ELISA and infectivity assay are shown in Fig. 1(a, b). Intracellular CA-p24 was not assessed at this stage due to the high level of variability seen in this parameter, possibly due to CA-p24 being produced prior to helicase knockdown or variability in the effectiveness of cell lysis.

**Fig. 1.**
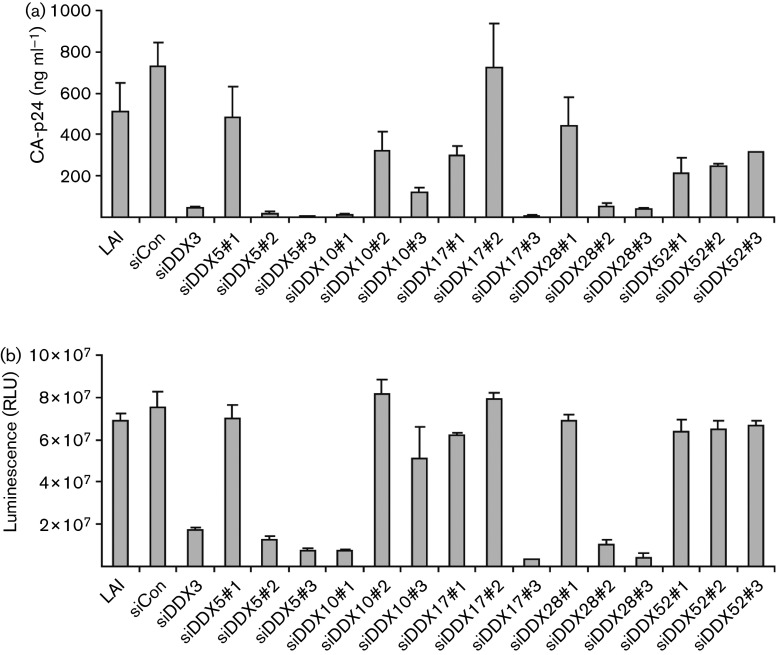
Effects of the siRNAs of interest on WT HIV-1 replication. HeLa cells in 24-well plates were sequentially transfected, first with the indicated siRNAs and 24 h later with pLAI. Then, 48 h later supernatant samples were harvested for (a) CA-p24 ELISA and (b) infectivity assay. The experiment was performed in triplicate and values represent mean±sd. LAI, Cells transfected with viral DNA alone; siCon, negative control siRNA; siDDX3, positive control; RLU, relative light units.

Compared with siCon, the pool of DDX3 siRNAs significantly decreased CA-p24 (Fig. 1a) production as expected from the published literature. Several siRNAs (siDDX5#2, siDDX5#3, siDDX10#1, siDDX10#3, siDDX17#3, siDDX28#2 and siDDX28#3) caused a reduction in CA-p24 equal to or greater than that seen with siDDX3. At the other extreme, three siRNAs (siDDX5#1, siDDX17#2 and siDDX28#1) had little or no effect on CA-p24 production.

Interestingly, the siRNA effects were generally greater on viral protein production than on infectious virus production. For example, the three siRNAs against DDX52 caused a modest reduction in CA-p24 production; however, there was little difference in the infectivity of the virions produced from these cells. Possible explanations for this include the siRNAs causing fewer virions to be produced, but for these virions to be more infectious. Alternatively, these results may represent a reduction in the amount of free CA-p24 or abnormal virions released from cells.

Only the DDX52 siRNAs gave a consistent phenotype with all three siRNAs. For the other four helicases, two of the three siRNAs significantly impacted on viral replication, as had been seen at all previous stages of screening. This difference in effect of siRNAs against the same target may represent differing levels of knockdown of the target protein achieved by the individual siRNAs.

To assess siRNA-mediated knockdown, Western blot analysis was performed on lysates of siRNA-treated HeLa cells. All the siRNAs against DDX5 (Fig. 2a), DDX10 (Fig. 2b), DDX17 (Fig. 2c) and DDX28 (Fig. 2d) successfully reduced helicase expression to a similar degree with little effect on β-actin levels. This did not correlate with the different effects of siRNAs on viral replication (Fig. 1). We hypothesize that a critical level of a helicase is required for optimal virus production and if an siRNA fails to reduce helicase expression below this level, virus production is not affected. The immunoblotting assays may not be sufficiently sensitive to observe these perhaps subtle but critical differences in protein expression.

**Fig. 2.**
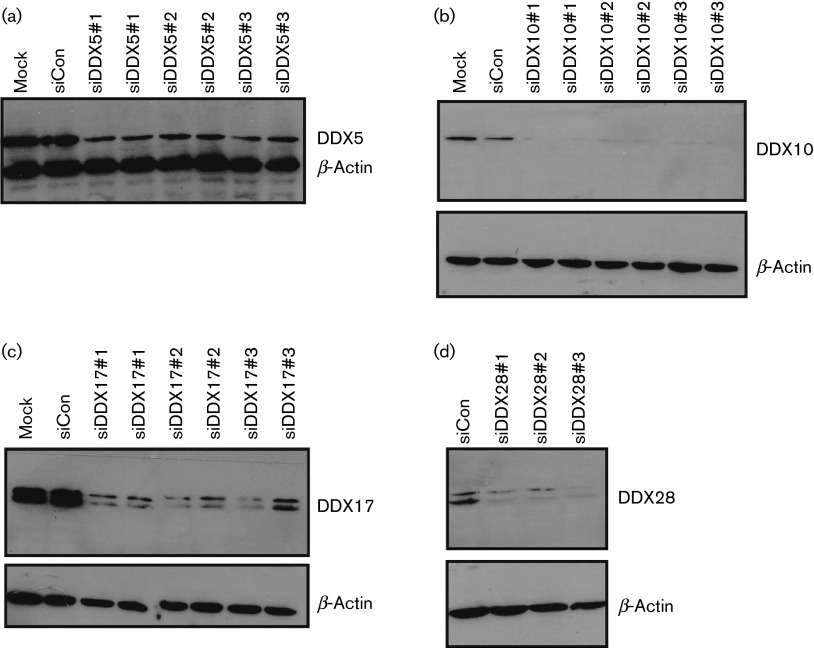
Western blots confirming the effect of the siRNAs on helicase expression levels. HeLa cells in 24-well plates were transfected with 20 pmol of the indicated siRNA using 1 µl Lipofectamine 2000. At 48 h post-transfection, cells were lysed using cell culture lysis reagent (Promega) and samples subjected to SDS-PAGE, transferred to nitrocellulose membranes and probed for (a) DDX5, (b) DDX10, (c) DDX17 or (d) DDX28 expression. All membranes were subsequently probed for β-actin to determine equal loading.

Due to the lack of a good antibody, a Western blot for DDX52 is not shown. With the exception of DDX28 the knockdown achieved with each siRNA is shown in duplicate as compared with the baseline expression of the helicase. The DDX17 blot (Fig. 2c) shows two protein bands as the DDX17 mRNA can be alternately translated from a non-AUG upstream start codon. Our three siRNAs reduced the expression of both transcriptional variants of DDX17 to a similar degree. The identities of the two protein bands detected by the DDX28 antibody are less clear, but may represent different isoforms or post-translational modification (Fig. 2d).

To rule out siRNA-induced cell death being non-specifically responsible for the effects seen on viral replication, we performed a luminescence-based cell viability assay (Cell-Titre Glo; Promega). This assay was performed in the absence of virus production, with puromycin included as a positive control for cell death. As can be seen in Fig. 3, none of the siRNAs caused the dramatic level of cell toxicity seen with puromycin; however, 10 of the 15 siRNAs did result in statistically significant higher levels of cell death than the control siRNA.

**Fig. 3.**
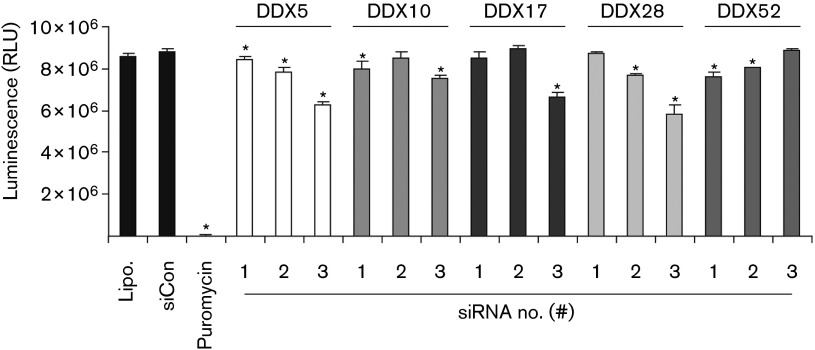
Viability assay. HeLa cells in half-area 96-well plates were transfected with 2.5 pmol of the indicated siRNAs using Lipofectamine 2000 (Lipo.). Puromycin (10 µg ml^−1^) was used as a positive control. At 72 h post-transfection, the luminescent-based viability assay was performed. The experiment was performed in triplicate and values represent mean±sd. *Statistically significantly different from control siRNA-treated cells (Student’s *t*-test, independent, two-tailed, *P*<0.05). RLU, Relative light units.

Comparing the results in Fig. 1 with those in Fig. 3 it is clear that in some cases the siRNAs that caused the greatest inhibition of viral replication also had the greatest effect on cell viability. There is, however, a considerable variation in the magnitude of these effects, e.g. siDDX5#2 reduced infectious virion production by ~80 % compared with siCon, whilst it only reduced cell viability by ~10 %. It is likely that the siRNAs which had the greatest effect on helicase expression also had the greatest effects on both viral replication and cell viability.

Overall, our siRNA library screen identified five helicases which when knocked down reproducibly reduced infectious virion production, suggesting a likely role in HIV-1 replication. Although our screen was performed in HeLa cells, all five helicases are thought to be expressed in cell types relevant to HIV-1 replication (Krishnan & Zeichner, 2004; Lin *et al.*, 2013; Valgardsdottir *et al.*, 2001; Whitney *et al.*, 2003).

Helicases with published roles in HIV-1 replication were not taken beyond the first round of screening. However, the roles of DDX5 and DDX17 in viral replication were published during the course of this work, and so they were included as further positive controls. These two proteins are highly related and can exist as homo- or heterodimers (Lamm *et al.*, 1996; Ogilvie *et al.*, 2003). Their proposed cellular functions include splicing, miRNA (microRNA) processing and transcriptional regulation (reviewed by Fuller-Pace, 2013). DDX17 has been shown to associate with HIV-1 Rev, with helicase knockdown leading to reduced CA-p24 release from infected cells, possibly due to an effect on Rev-mediated nuclear export (Naji *et al.*, 2012; Yasuda-Inoue *et al.*, 2013). Further proposed roles for DDX17 include viral RNA packaging, Gag–Pol frameshifting and regulation of viral RNA stability (Chen *et al.*, 2008; Lorgeoux *et al.*, 2013; Zhu *et al.*, 2011). Overall, our work supports the previously recognized phenotype that DDX17 knockdown significantly reduces HIV-1 virion production.

DDX5 has also been shown to interact with Rev (Yasuda-Inoue *et al.*, 2013). However, knockdown of DDX5 gives complicated results, with three different studies giving three different results ranging from reduced CA-p24 production, supported by our results, to a significant increase in both CA-p24 and infectivity (Lorgeoux *et al.*, 2013; Naji *et al.*, 2012; Zhou *et al.*, 2013). The main differences between these studies were the cell type and transfection protocol used. It has been speculated that knockdown of DDX5 causes an increased formation of DDX17 homodimers, indirectly affecting viral replication (Naji *et al.*, 2012). Indeed, it has previously been suggested that the relative abundance of DDX5 and DDX17 influences whether homodimers or heterodimers are formed (Ogilvie *et al.*, 2003). It has also been suggested that knockdown of one of the helicases causes a compensatory increase in the expression of the other (Shin *et al.*, 2007). This complex interaction makes the interpretation of DDX5 or DDX17 knockdown experiments difficult. Different basal levels of helicase expression in different cell types and different knockdown efficiencies may contribute to the different results seen. Further experiments, including siRNA rescue assays and the assessment of helicase dimerization state, are needed to further understand the relative contributions of DDX5 and DDX17 to the HIV life cycle.

Comparison of the results of our siRNA library screen with previously published genome-wide siRNA screens reveals only one overlap: DDX10 (Brass *et al.*, 2008). This is perhaps unsurprising given the low overall level of commonality seen in the three genome-wide screens. In addition to being identified in the Brass *et al.* (2008) screen, DDX10 expression has been shown to be significantly upregulated in response to HIV-1 infection (Krishnan & Zeichner, 2004). The same experiment also showed DDX52 to be upregulated during viral replication. This raises the possibility that HIV-1 induces the expression of helicases required for optimal viral replication. The cellular functions of DDX10 and DDX52 have not been fully elucidated, but they are both proposed to be involved in ribosomal synthesis (Savitsky *et al.*, 1996; Venema *et al.*, 1997). Even less is understood about DDX28 – it is thought to be a component of mitochondrial DNA nucleoids, but its cellular function remains to be elucidated (Bogenhagen *et al.*, 2008; Valgardsdottir *et al.*, 2001). It is possible that the normal cellular functions of these RNA helicases are required for optimal HIV-1 replication, e.g. to enable viral protein expression. However it is also plausible that the virus has hijacked the helicases to fulfil novel roles not seen in uninfected cells.

Experiments to clarify the roles of these helicases are ongoing, including whether the effects can be reproduced in T-cells and at what stage of the viral life cycle the helicases are being utilized.
